# Childhood Occurrence of Pemphigus

**DOI:** 10.5005/jp-journals-10005-1434

**Published:** 2017-06-01

**Authors:** Raju U Patil, Rajesh T Anegundi, Kumar R Gujjar, KR Indushekar

**Affiliations:** 1 Professor and Head, Department of Pedodontics and Preventive Dentistry, Sinhgad Dental College & Hospital, Pune, Maharashtra, India; 2 Professor and Head, Department of Pediatric Dentistry, SDM College of Dental Sciences & Hospital, Dharwad, Karnataka, India; 3 Senior Lecturer, Department of Pediatric Dentistry, SEGi University, Kota Damansara, Selangor, Malaysia; 4 Professor and Head, Department of Pedodontics and Preventive Dentistry, Sudha Rustagi College of Dental Sciences & Research, Faridabad Haryana, India

**Keywords:** Acantholysis, Autoimmune, Blistering disease, Corticosteroids, Pemphigus.

## Abstract

Pemphigus is a chronic mucocutaneous disease that initially manifests in the form of intraoral blisters which spread to other mucous membrane and skin. This study describes an unusual case of chronic generalized childhood pemphigus disease in an 11-year-old girl, who presented with multiple vesicles all over her body. Such a condition is seen more often in older people rather than children. It is crucial for dental professionals to be familiar with the diagnosis of bullous skin diseases in children and adolescents, especially in its initial stages in order to prevent the serious consequences and morbidity. The article highlights clinical presentation, histopathology, and successful management strategies useful for pediatric dental practice.

**How to cite this article:**

Patil RU, Anegundi RT, Gujjar KR, Indushekar KR. Childhood Occurrence of Pemphigus. Int J Clin Pediatr Dent 2017;10(2):196-200.

## INTRODUCTION

Blistering diseases are facing the danger of being finished, since our understanding of the pathogenesis and therapeutic approaches are undergoing a major revision. A wide spectrum of skin disorders can manifest as a blistering process. A blister is an event associated with tissue injury and fluid accumulation within a specific layer of skin due to either genetic mutations or autoimmune response. Blisters can also occur secondary to bacterial/viral infections, chemical/physical burns or skin necrosis/dermatitis.^1,2^ Here, the focus of interest is a bullous dermatoses in the child based on a case of pemphigus vulgaris (PV).

The PV is an autoimmune blistering disease of elderly (3rd-5th decade), which was previously fatal before the advent of steroid therapy, mainly due to dehydration or secondary systemic infection.^3,4^ The PV is characterized by the presence of circulating autoantibodies immuno-globulin G against desmogleins 3^5,6^ which result in loss of cell to cell adhesion and blister formation that rupture and progress to form painful erosions.^4^ The PV in children aged less than 12 years is known as childhood PV and in those aged between 12 and 18 years as juvenile PV. Data on incidence and prevalence of childhood PV are scarce because in literature only a few cases are reported. In a study, children aged less than 15 years accounted for 3.7% of cases.^7^ Several environmental factors, medications, and acantholytic substances superimposed on genetic predisposition may play a role in the onset of this disease in children.^8^

## CASE REPORT

**Figs 1A and B:**
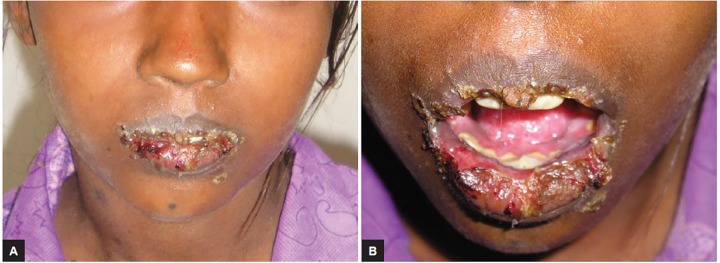
Multiple crushed lesions with superficial erosions on lips

**Figs 2A and B:**
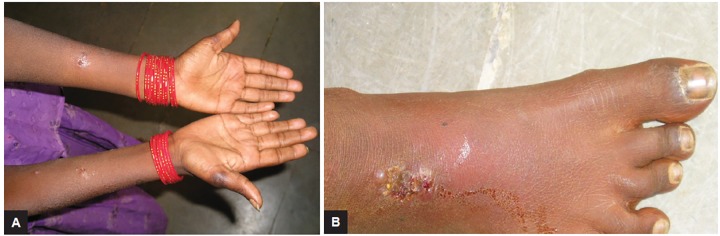
View of lesions on the limbs

**Figs 3A and B:**
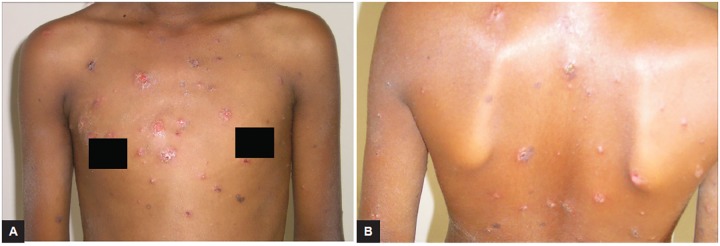
View of wide spread lesions all over the body

An 11-year-old girl presented to the department of pedi-atric dentistry, with a complaint of multiple eruptions and blisters all over the mouth, which increased in size gradually over a period of 2 to 3 months and ruptured to form a crusty erosive surfaces with watery discharge (Fig. 1). Later, similar sores appeared on limbs, trunk, and the genital area which were painful and led to considerable discomfort (Figs 2 and 3).

Entire oral mucosa including the tongue was eroded and erythematous, causing extreme discomfort and pain during eating. There was no history of any drug intake during the past 6 months nor any systemic condition identified. The child presented with such a condition for the first time and there was no such disorder noted in the family. Nikolsky’s perilesional sign was positive.

**Fig. 4:**
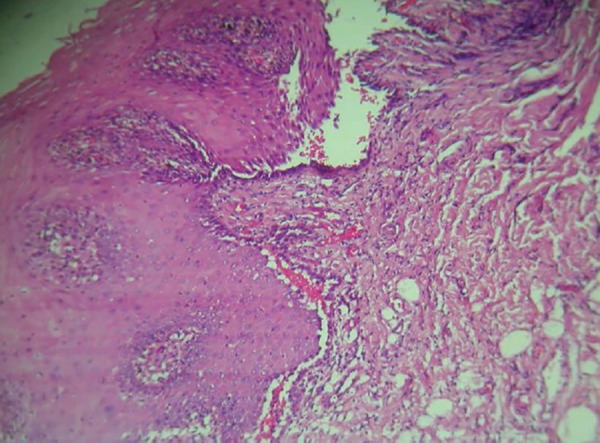
Acantholysis and suprabasilar separation

**Fig. 5:**
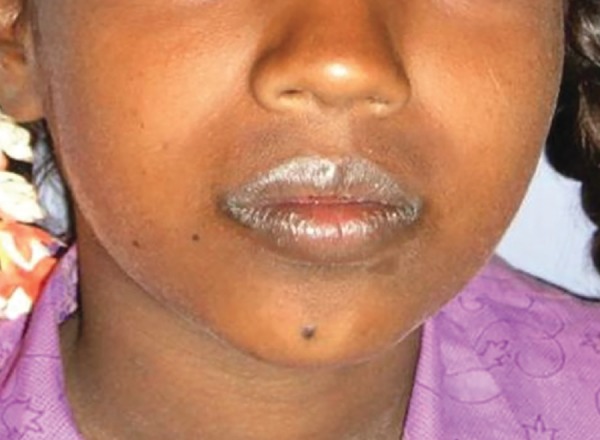
Posttreatment view

**Figs 6A and B:**
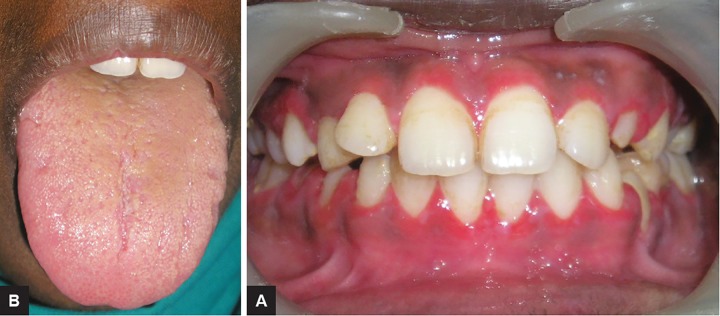
Lesions disappear following standard treatment regime

**Figs 7A to C:**
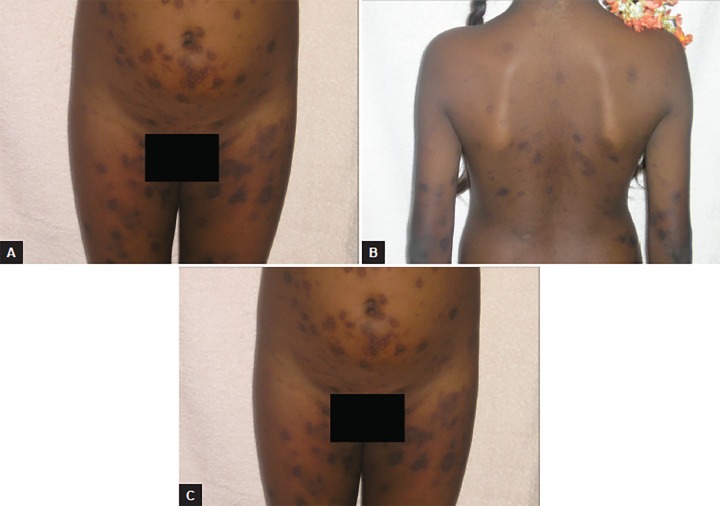
Healing of lesions all over the body

The girl was hospitalized in the medical unit and comprehensively managed with the help of a dermatologist (Tables 1 and 2). Direct immunofluorescence was positive and perilesion biopsy containing intact lesion, revealed Tzanck cells, intraepidermal blister and suprabasilar acantholysis (Fig. 4). The connective tissue stroma showed dense mononuclear infiltration. A significant improvement in the condition was observed after 3 to 4 weeks following the standardized steroid treatment regime (Figs 5 to 7).

## DISCUSSION

Unusual childhood occurrence, though quick response to treatment, however potentially life-threatening nature with substantial morbidity, justifies its consideration in routine dental practice. These chronic recurrent and painful lesions interfere with the daily activities of life, such as eating, drinking, talking, and personal relationships.^9^ Pediatric dentists have the unique opportunity since initial lesions occur in the oral cavity and complete remission is possible only with early diagnosis.^10^

Prompt diagnosis and early initiation of aggressive therapy can combat the malignant course of disease in children. The treatment strategies should be based on the understanding of underlying pathogenic processes and recurrence^3,11-14^ (Tables 3 and 4). Systemic corticosteroids and immunosuppressive therapy are the mainstay treatments for PV. Apart from steroids, adjuvant therapies include azathioprine, mycophenolate mofetil, dapsone, and rituximab in refractory cases.^4,7,8^ These modern therapies can effectively reduce the circulating antibodies, allowing patients to lead a normal life. Adverse effects associated with long-term use of steroids, such as weight gain, menstrual irregularities, growth retardation, osteoporosis, and hormonal disturbances in adolescence^4,5^ have always led to the search for newer steroid sparing and novel avenues for eradication of blisters at the molecular level.^1,2^ As we probe deeper into molecular aspects of the disease, our understanding of the patho-genesis begins to gain focus, offering new novel, and improved methods of therapy or even an opportunity to achieve a cure, which should mark the end of an era of blistering diseases.

**Table 1:** **Table 1:** Systemic treatment regime

*Drugs*		Dose, route, and duration*		*Action*	
Dexamethasone		0.5 mL Inj IM (50-100 mg) 3 to 4 weeks		Modification of immune response (immunosuppresion)	
Roxithromycin		150 mg Tab BID - 2 to 3 week		Antibacterial for secondary infection	
Prednisolone		10-20 mg Tab tapering to 5 mg		Anti-inflammatory and modification of immune response	
		BID - 2 to 3 months			
Hematopoietics		Oral capsule OD - 1 month		Nutritional supplement	
NaCl saline		IV fluid		Electrolytic balance	

*Minimum duration is 3 to 4 weeks, may be extended depending on response and recurrence; IM: Intramuscular injection; BID: Twice (two times) a day; OD: Once daily; IV: Intravenous

**Table 2:** **Table 2:** Topical treatment regime

*Drugs*		*Dose, route, and duration’*		*Action*	
Triamcinolone		Local application for more than 3 weeks		Potent anti-inflammatory and alters immune response	
Silver sulfadiazine and chlorhexidine		Local application for more than 2 weeks		Broad spectrum antimicrobial	
Gentamycin with propyl salicylic acid		Local application for more than 2 weeks		Prevents secondary infections	
Saline compresses over erosive lesions		Local application for more than 2 weeks		For soothing effect and control of edema	
Chlorhexidine		Oral gargle for more than 3 weeks		Oral antimicrobial	

**Table 3:** **Table 3:** Protocols for preventing recurrence^3,12-14^

		Maintaining healthy diet and weight	
		Avoiding sunlight and friction of body folds	
		Keeping flexural areas clean and dry	
		Wearing cool garments with absorbent pads	
		Regular evaluation of secondary infections	
		Systemic antibiotics, such as tetracycline and erythromycin	
		Topical use of antibacterial creams, such as benzyl peroxide	
		Long-term low-dose steroid maintenance therapy	
		Controlling side effects of long-term steroids	

**Table 4:** **Table 4:** The bullous management portfolio^5,11-14^

		Gold line mainstay of therapy - Steroids (Systemic prednisone 1 mg/kg/day and topical triamcinolone)	
		Broad-spectrum antibiotics for control of secondary infections Improving the general health and hygiene of the patient (Fluid replacement, electrolytic balance, and multiple vitamins/ minerals)	
		Symptomatic relief of pain, discomfort, burning, and itching (Paracetamol, astringents, and aluminium acetate)	
		Steroid sparing immunosuppressant and adjuvants (Mycophenolate mofetil, tracolimus, azathioprine, dapsone, retenoids methotrexate, cyclophosphamide, gold, cyclosporine, and chlorambucil)	
		Newer vistas - Plasmapheresis, intra venous immunoglobulins, anti-B cell monoclonal antibodies, CO_2_ laser vaporization, dermabrasion, proteinase inhibitors, chimeric molecules, cholinergic agonists, etc.	
